# Comparison and Characterisation of Regenerated Chitosan from 1-Butyl-3-methylimidazolium Chloride and Chitosan from Crab Shells

**DOI:** 10.1155/2015/874316

**Published:** 2015-05-19

**Authors:** Saniyat Islam, Lyndon Arnold, Rajiv Padhye

**Affiliations:** Centre for Advanced Materials and Performance Textiles, RMIT University, Melbourne, VIC 3056, Australia

## Abstract

Chitosan is a biopolymer derived from chitin which is naturally occurring in the exoskeleton of crustaceans. This paper reports dissolution and regeneration of chitosan by directly dissolving in an ionic liquid solvent, 1-butyl-3-methylimidazolium chloride (BMIMCl). This will provide an ideal platform to solubilise these kinds of polymers to achieve the dissolution. The current study dissolved chitosan from crab shell utilising BMIMCl as a solvent and characterised the resultant regenerated polymer. The regenerated chitosan showed increased hydrogen bonding when characterised by Fourier transform infrared (FTIR) spectral analysis. In addition, the study also compared the characteristics of regenerated and generic chitosan. The regenerated chitosan was also evaluated for antimicrobial properties and showed to possess antibacterial features similar to the commercial grade. This method can be utilised in future for blending of polymers with chitosan in a dissolved phase.

## 1. Introduction

**Figure 1:**
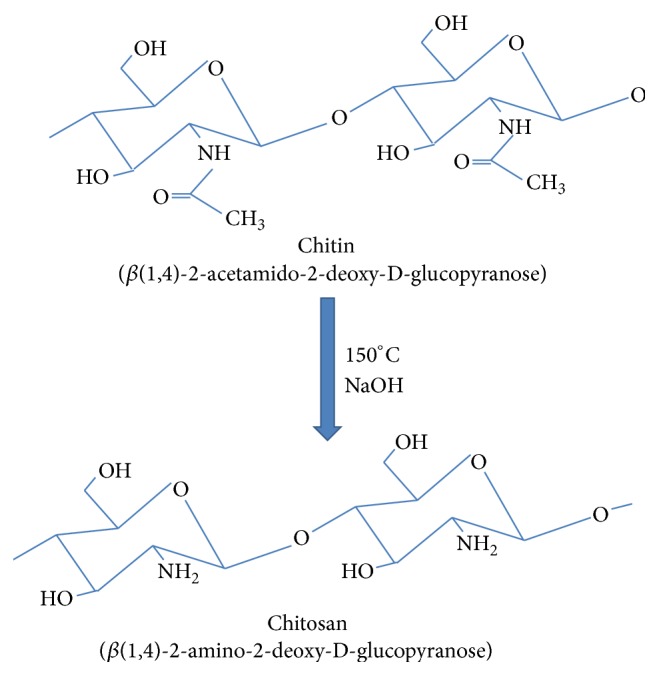
Deacetylation of chitin to obtain CHT [1].

Chitosan (CHT) is a linear polysaccharide composed of randomly distributed *β*-(1-4)-linked D-glucosamine (deacetylated unit) and N-acetyl-D-glucosamine (acetylated unit) [1–9]. Chitosan is formed commercially by deacetylation of chitin, which is the compositional element in the exoskeleton of crustaceans such as crabs, shrimps, and lobsters. Preparation of chitosan from chitin is given in Figure 1.

CHT has three reactive groups and they are primary (C-6) and secondary (C-3) hydroxyl (–OH) groups and the amino-NH2 (C-2) group in each repeat of the deacetylated unit of chitin. Thus it is polycationic in nature. The antimicrobial activity of CHT and its derivatives has been well proven in past studies but the mechanism of the antimicrobial action is yet to be discovered. The most acceptable interpretation is that the anionic cell surface of the microbes interacts with the cationic CHT, causing extensive cell surface alterations and damage. This leads to inhibition of the metabolism of the cell and results in its death [10]. So far it is considered that CHT acts as a biocide for some microbes and as a biostatic agent for others [11]. Other than its antimicrobial activity, CHT has been extensively studied for other applications such as photography, cosmetics, artificial skin, dressings and wound healing, food and nutrition, wastewater treatment, and dyeing and printing [12]. CHT also has been investigated for its capability of forming continuous films [13–16].

**Figure 2:**
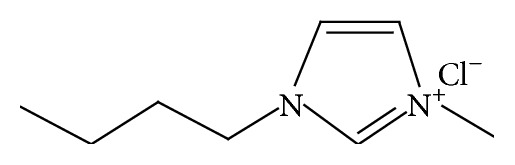
Chemical structure of BMIMCl.

Ionic liquids (ILs) can be derived from a wide variety of complex compounds such as salts of imidazolium, ammonium, pyridinium, isoquinolinium, sulfonium, phosphonium, and pyrrolidinium [17]. Ionic liquids based on monosubstituted to pentasubstituted imidazolium ions are favourable species for investigation in textiles because of their air and water stability, their wide range of liquidity, the fact that they remain liquid at room temperature, and their relatively favourable viscosity and density characteristics [18, 19]. This paper reviewed the developments and applications of one significant IL with application for textiles and polymers, specifically, 1-butyl-3-methylimidazolium chloride (BMIMCl) which has the chemical structure shown in Figure 2 [6].

## 2. Materials and Methods

Practical grade CHT from crab shells (Brookfield viscosity 200 cP, 1% in 1% acetic acid, degree of deacetylation >85%, and molecular weight 190,000–>375,000, which is based on the viscosity of 200–2000 mPa·s) was sourced from Sigma-Aldrich Pty Ltd., Australia, and used as received for the experiments. BMIMCl was also sourced from Sigma-Aldrich, Australia, and used without any modification.

### 2.1. Regeneration of CHT from BMIMCL

To dissolve the CHT, 1 g of CHT was mixed with 20 mL of BMIMCl, heated, and stirred at 100°C~130°C to obtain a 5 wt% completely homogeneous CHT solution. The dissolved CHT could be regenerated in water as BMIMCl is completely miscible with water in any ratio. This can be done by pouring the viscous solution into deionised water and coagulated several times. Regenerated CHT was obtained by casting the viscous solution between two circular microscopic slides and then soaked in water bath to allow BMIMCl diffusion from the films. During this process, water was changed several times to visually confirm that the BMIMCl had been removed completely from the sample; that is, no diffusion of BMIMCl could be observed. This was further confirmed by FTIR spectroscopy and discussed in the results section. After washing with deionised water several times the regenerated CHT films were dried in a vacuum oven to get flat blend films.

### 2.2. FTIR Spectroscopy

The FTIR spectra were collected by a spectrometer (PerkinElmer Spectrum-400) to determine the functional groups of the fibres/fabrics. The number of scans per sample was 8 and the FTIR spectra were collected in the wave number region of 4000–650 cm^−1^. The spectra indicated the absorbance/transmittance of a material as a function of wave number.

### 2.3. Antibacterial Testing

A modified AATCC TM 100-2004 (clause 10.2) test method was followed to assess the antibacterial properties of samples.* Escherichia coli* (*E. coli*) strain-ATCC 11229, a gram-negative bacterium, was used as the test organism. Bacterial inoculums were prepared to obtain a suspension in an exponential growth of 10^8^ colony forming units (CFU) mL^−1^ in 5 mL of modified tryptone-soy nutrient broth. Fabrics dyed in the absence of CHT were used as negative control samples. Antibacterial tests were conducted on each sample individually as outlined by Zhang et al. [20]. In brief, 1 mL of inoculum was added to a 0.1 gm of CHT in a conical flask. 100 mL of distilled water was added to the flask and this was shaken vigorously for 1 minute. From this solution, a series of dilutions were prepared as 10^0^, 10^1^, 10^2^, and 10^3^ times with sterile distilled water. The dilutions were then plated in triplicate and incubated for 18 hours at 37°C. After incubation, the control plates exhibiting 30–300 colony forming units were taken as reference. Test plates of a similar dilution were compared. The percentage reduction of bacteria was calculated by the following equation [21]:

(1) $$\begin{array}{c} \text{Reduction}\;\;\text{in}\;\;\text{CFU}\%=\frac{\left(X-Y\right)}{X}\times 100\%, \end{array}$$

where *X* is the average number of bacterial colonies in the control and *Y* is the average number of bacterial colonies in the presence of CHT.

## 3. Results and Discussion

**Figure 3:**
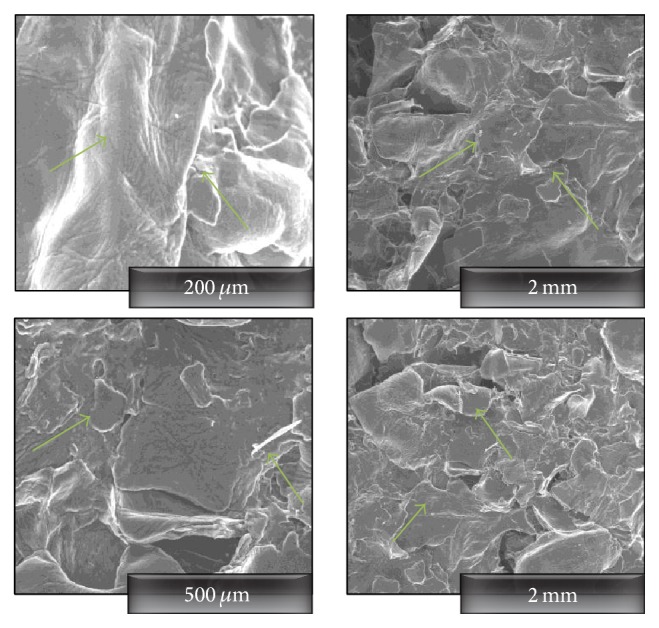
Appearance of a scaly structure at various locations of the CHT film regenerated from BMIMCl solution.

Regenerated CHT from BMIMCl was characterised for its morphological changes as dissolution and regeneration occur. The appearance can be distinguished as defragmented scaly structure. It is noticeable that although the film visibly seems uniform, the scaly structure is apparent throughout the film region inspected.  Figure 3 shows the morphological structure of a typical CHT film prepared from BMIMCl at different locations within the film.

FTIR studies of CHT from crab shells (sourced from Sigma-Aldrich) and CHT of the same regenerated from BMIMCl solution were characterised for any chemical and structural derivatisation of CHT in both forms.

**Figure 4:**
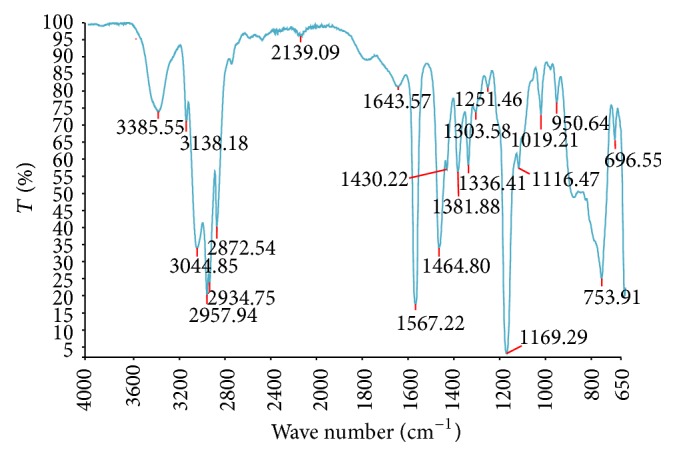
FTIR spectrum of BMIMCl.

The normalised spectrum obtained from FTIR spectroscopy of BMIMCl is shown in Figure 4 [6]. The functional groups present and their respective wave numbers (cm^−1^) and intensities (%T) confirm the chemical structures given in Figure 4. The peak stretching at wave number 3385 (cm^−1^) corresponds to N–H (2° amines) bonding. The wave numbers from 3138 (cm^−1^) to 2872 (cm^−1^) refer to O–H stretching. The bending at 1643 (cm^−1^) is indicative of C=N and C=C bonding and peak ranging between 696 (cm^−1^) and 1567 (cm^−1^) corresponds to stretching C–C, C–O, or C–N bonds.

**Figure 5:**
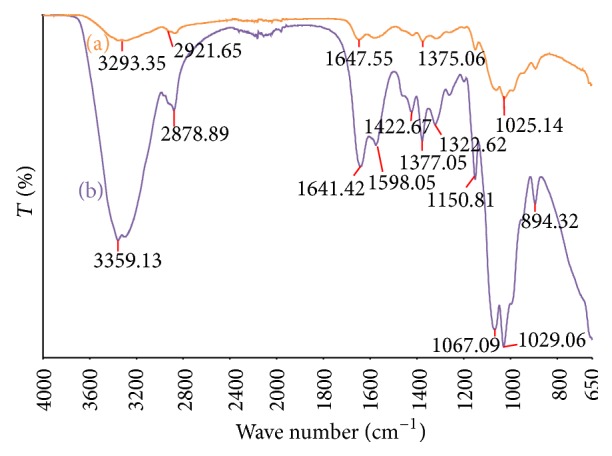
FTIR spectra of (a) CHT from crab shell and (b) CHT regenerated from BMIMCl.

FTIR spectra of CHT from crab shells and CHT regenerated from BMIMCl are shown in Figure 5.

Both spectra show similar patterns; however, the regenerated CHT produced sharper peaks at 3359 cm^−1^ and in the 1030–1155 cm^−1^ regions. The transmittance peak in this area indicates stretching of the O–H and N–H bonds at 3359 cm^−1^ and C–O bonds at 1030–1155 cm^−1^, respectively. In addition, absorption peaks in the 2880 cm^−1^ region, around 1550–1590 cm^−1^ and 1400 cm^−1^, correspond to C–H stretching, amine groups-(NH_2_), and carboxyl groups (–COO^−^), respectively [22]. A band at 3359 cm^−1^ corresponds to the combined peaks of the NH_2_ and O–H group stretching vibration in CHT. The band at 1641 cm^−1^ is attributed to the CO–NH_2_ group. The 1598 cm^−1^ transmittance peak of the –(NH_2_) bending vibration is sharper than the peak at 1641 cm^−1^, which shows the high degree of deacetylation of the CHT. A shift from 3293 to 3359 cm^−1^ is shown and the peak is sharper in the regenerated CHT, which indicates that the hydrogen bonding is enhanced [23].

The intensities of the (CO–NH_2_) band at 1641 cm^−1^ and the (NH_2_) band at 1598 cm^−1^, which can be observed clearly in pure CHT, increase dramatically, and two new sorption bands at 1422 cm^−1^ and 1322 cm^−1^ appear, which show asymmetrical C–H bending of the CH_2_ group. Thus it is postulated that the halide (Cl^−^) of BMIMCl interacts with the ammonium groups of CHT, which serves to enhance both the inter- and intramolecular interaction in regenerated CHT [24]. To summarise, 3429 cm^−1^ (O–H stretching overlapping the N–H stretching), 2921 and 2878 cm^−1^ (C–H stretching), 1641 cm^−1^ (amide II band, C–O stretching of the acetyl group), 1598 cm^−1^ (amide II band, N–H stretching), 1485–1380 cm^−1^ (asymmetrical C–H bending of the CH_2_ group), and 1029 cm^−1^ (O–bridge stretching) of the glucosamine residue [25] of CHT can be characterised using FTIR spectroscopy. This also indicates that no derivatisation occurred during the dissolution and regeneration stages. In addition, the fingerprint bands of BMIMCl are absent in the regenerated CHT when compared to Figure 4 which confirms complete removal of BMIMCl in the washing process. The assignments of the bands are listed in Table 1.

### 3.1. Calculation of Degree of Deacetylation from FTIR Spectra

The degree of deacetylation (DD) is one of the most important chemical parameters capable of influencing the performance of CHT in many of its applications [25–28], one of them being antimicrobial efficacy. Several analytical techniques were developed for DD determination using IR spectroscopy. Quite a few absorption band ratios, such as A1560/A2875, A1655/A2875, A1655/A3450, A1320/A3450, A1655/A1070, A1655/A1030, A1560/A1160, A1560/A897, and A1320/A1420, have been suggested by several researchers [29] to determine DD by FTIR spectroscopy. Since the CHT used for the current study has DD of >85% (supplier's information), two calculation methods were used to determine DD of the CHT and regenerated CHT [29]:

(2) $$\begin{array}{c} \frac{A_{1560}}{A_{2875}}=0.0125\times \text{DD}+0.2\;\left(R^{2}=0.99\right). \end{array}$$

See [30]:

(3) $$\begin{array}{c} \text{DD}=118.883-\left(40.1647\times \;\frac{A_{1655}}{A_{3450}}\right). \end{array}$$

See [31].

Equation (2) produces a calculated DD value of 70% and (3) suggests a calculated DD of 68% and both the values are lower than the specification provided by the manufacturer. However, for the regenerated CHT samples, the calculated values of DD are 89% and 84% which closely matches the supplier's specification. This increase can be attributed to higher hydrogen bonding caused by dissolution and regeneration phenomenon.

### 3.2. Temperature-Dependent Dissolution Phenomena

**Figure 6:**
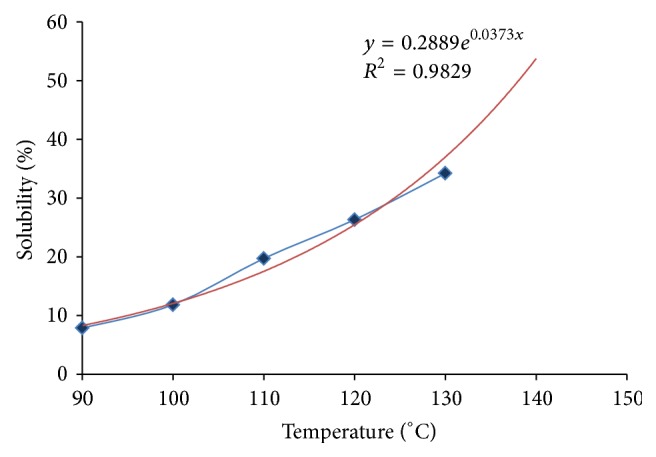
Temperature-dependent dissolution of CHT (blue) in BMIMCl.

The dissolution phenomenon of CHT was investigated and the total amount dissolved in a particular amount of BMIMCl was calculated as a function of time elapsed to dissolve the sample. A graph was plotted (as shown in Figure 6) to show the dissolution.

Recent studies [32, 33] compared dissolution polymers in a variety of ionic liquids. However, the time it takes to reach the maximum yield was reported differently in several studies [34–36]. As can be seen from Figure 5, the dissolution temperature for CHT started from 90°C and the maximum yield was reached at 130°C. In addition, the time of dissolution was observed to be much less (4 hours) than in previous studies. This anomaly can be explained by the elevated temperature as well as the fineness and form of CHT samples used (powder or larger scales). The maximum yield was observed to be approximately 5% on the weight of BMIMCl used, which is in good agreement with previous studies [35, 36]. An equation for the dissolution of CHT in BMIMCl is derived by plotting an exponential curve fit. The generalised equation for exponential scale is *y* = *e* ^*mx*^:

(4) $$\begin{array}{c} \text{For}\;\;\text{CHT}\;\;\text{dissolution},\;\;y=0.2889e^{0.0373x}\;\left(R^{2}=0.9829\right), \end{array}$$

where *m* is the dissolution rate of the polymer.

### 3.3. Antibacterial Evaluation

**Figure 7:**
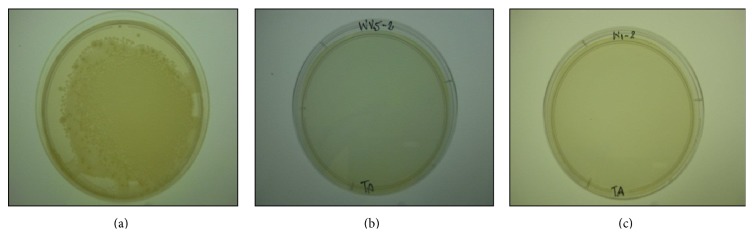
(a) Control showing complete growth of bacteria and (b) CHT from crab shell showing no growth and (c) CHT regenerated from BMIMCL showing no growth.

Figure 7 shows the antibacterial effect of the two CHT samples against the control. As there was no growth observed the antibacterial activity was excellent for both sources of CHT samples.

The outcome of the antibacterial testing conducted is summarised in Table 2. As the samples were repeated in triplicate the results are averaged for tabulation.

## 4. Conclusion

A comparative study of CHT from crab shells and regenerated of the same from BMIMCl showed that CHT can be dissolved and regenerated in BMIMCl without any decrease in the functional attributes such as antibacterial behaviour. The commercial grade of CHT used in this study can usually be dissolved by weak acids; however, these media might not be suitable for other polymers when solution blending is required. BMIMCl provides an ideal medium for solution blending of different natural polymers with CHT which has been shown in this study.

## Figures and Tables

**Table 1:** FTIR bands of CHT and regenerated CHT with assignments.

Wave number (cm^−1^)	Vibrational assignments
3359	O–H and N–H stretch
2921 and 2878	C–H stretch
1641	Amide II and C–O stretch of acetyl group
1598	N–H stretching from amide I and amide II
1485–1380	Asymmetrical C–H bending of the CH_2_ group
1067	Asymmetric stretch C–O–C and C–N stretch
1029	O–bridge stretching of the glucosamine residue

**Table 2:** Reduction in CFU% according to (1).

Sample	Plate count	Reduction in CFU%
Control	Uncountable	0
CHT from crab shell	0	100%
Regenerated CHT	0	100%
